# Preventing Technostress Through Positive Technology

**DOI:** 10.3389/fpsyg.2018.02569

**Published:** 2018-12-17

**Authors:** Eleonora Brivio, Fulvio Gaudioso, Ilaria Vergine, Cassandra Rosa Mirizzi, Claudio Reina, Anna Stellari, Carlo Galimberti

**Affiliations:** ^1^Department of Psychology, Centro Studi e Ricerche di Psicologia della Comunicazione, Università Cattolica del Sacro Cuore di Milano, Milan, Italy; ^2^Department of Psychology, Università Cattolica del Sacro Cuore di Milano, Milan, Italy

**Keywords:** technostress, organizational safety culture, Positive Technology, Enterprise 2.0, work well-being

## Introduction

Over the past decade, the workplace has experienced significant changes as a result of Information and Communication Technologies (ICTs) and the subsequent digital transformation (Mcafee, 2006; Matt et al., 2015). Such technological, cultural, and organizational changes have redefined business models and competition. As evidenced by the shift from the Enterprise 1.0 to the Enterprise 2.0 business models, ICTs offer companies increased productivity and efficiency (Bilbao-Osorio et al., 2013). At the same time, introduction of ICTs can pose a threat to both a company and its employees through misuse, abuse, and overuse, resulting in technostress (Gaudioso et al., 2017). This emerging risk seems to have become more evident in the past 10 years, as a consequence of the 2008 economic crisis. This difficult and challenging economic context was demonstrated to have negatively impacted workers' mental health on its own, due to the workers' perception of the crisis, lack of social support, and increased job stress (Giorgi et al., 2015; Mucci et al., 2016). The economic crisis has had two paradoxical effects that indirectly may have contributed to the raise of technostress. On the one hand, the crisis reduced the number of total worked hours, reducing the resources needed. On the other hand, at the same time, it increased the pressure on the workers: corporations reduced available personnel—and thus increased tasks and activities on those remaining—and introduced new technologies to support their employees, who are required to deal with a higher work load and with managing new and more complex flows of information. This article aims to present the technostress construct, and propose how Positive Technologies (Riva et al., 2012) can help prevent technostress, and promote positive work experiences and general well-being through an effective organizational safety culture (Galimberti, 2014; Galimberti et al., 2016).

## Technostress

Technostress was first conceptualized in the early 1980s as “a modern disease of adaptation caused by the inability to cope with new technologies in a healthy manner” (Brod, 1984), which can result into non-acceptance of ICTs or excessive identification with the new technologies, resulting in both anxiety and stress. Today, technostress is considered to be multidimensional, and it is defined as “a negative psychological state associated with the use or the “threat” to use new technologies,” which leads to “anxiety, mental fatigue, skepticism, and sense of ineffectiveness” (Salanova et al., 2007). The fundamental dimensions to technostress include:

- Techno-anxiety: the use of computers or ICTs that generates fear, apprehension, and agitation in the user; it includes feelings of uncertainty resulting when a person is required to carry out an action using a ICT (e.g., pressing a button), and the related fear of losing information (Salanova et al., 2013).
- Techno-addiction: related to workaholism, it appears when an individual is unable to disconnect from work-related ICTs (e.g., phone, computer, etc.), therefore continuing to, often compulsively, perform work-related functions outside of normal business hours (Schaufeli et al., 2008); it can cause disconnection anxiety—the fear of being detached from the ICT device and information it provides (Elhai et al., 2016). It also manifests itself in an individual's behavioral patterns, such as constant anticipation of notifications, lack of control and/or difficulty in refraining from using ICTs, conflicts with other activities or tasks, and negative reactions to interrupted ICT use (Salanova et al., 2013).
- Techno-strain: perceived stress experience resulting from the use of new information technologies (Salanova et al., 2013).

Research shows that many factors contribute to technostress (Ragu-Nathan et al., 2008), including techno-invasion, techno-overload, techno-complexity, techno-insecurity, and techno-uncertainty. These stressors may have impact both at private and organizational levels. Techno-invasion, for example, is defined as constant connectivity, without boundaries of space and time, which maintains that employees are continuously available to work requests (Tarafdar et al., 2007; Ragu-Nathan et al., 2008; Gaudioso et al., 2017). Together with techno-addiction, techno-invasion entails that work-related tasks may spill into the worker's private life, endangering their work-life balance. At organizational levels, communication information overload (or techno-overload) results from employees' receipt of information from multiple channels simultaneously. This information can be difficult to manage, as it may be unclear how to prioritize or best use the information received (Tarafdar et al., 2007; Gaudioso et al., 2017). Another contributing factor is techno-complexity, the unpleasant feeling that the new ICTs are multifaceted and require tremendous effort to understand. Techno-insecurity is the perception that ICTs and the constant need to remain up-to-date can threaten an individual's job (Tarafdar et al., 2007). Lastly, techno-uncertainty causes perceived instability, due to the evolving nature of the work, and associated processes as well as constant introduction of new ICTs (Tarafdar et al., 2007).

Other contributing factors include: lack of support during testing, implementation, and use of the ICTs adopted by the company; discomfort and fatigue resulting from multitasking, as ICTs allow for completion of more tasks in a lesser amount of time (Ragu-Nathan et al., 2008); frequent interruption of assigned tasks due to the ongoing stream of communication (Mark et al., 2008). These stressors, together with a lack of personal coping mechanisms, create technostress in the work environment, placing both physiological and psychological consequences on employees. Proven physiological symptoms of technostress include fatigue (Salanova et al., 2007), irritability, insomnia (Porter and Kakabadse, 2006); psychological symptoms include frustration and perceived increased level of mental load and time pressure (Mark et al., 2008), skepticism, sense of ineffectiveness (Salanova et al., 2007), and reduction in job satisfaction and employee commitment, productivity, and work-life balance (Tarafdar et al., 2007). Technical and organizational support (Nelson, 1990), employees' involvement in the ICT implementation phase (Brod, 1984), and appropriate communication management (Galimberti, 2014; Galimberti et al., 2016) allow for decreased technostress emergence in organizations, as well as encourage greater well-being and productivity.

## Organizational Safety Culture

Because technological development and advancement are common in a multitude of organizations, companies must take technostress into consideration to care for their employees and thus their performance. Organizational culture refers to a set of processes, professional practices, explicit, and implicit rules, regulation, conventions, and shared ways of thinking within an organization. When these elements are linked to risk and safety in the workplace, they contribute to define a specific organizational safety culture (Galimberti, 2014). More specifically, von Thaden and Gibbons (2008) define safety culture as

“the enduring value and prioritization of worker and public safety by each member of each group and in every level of an organization. It refers to the extent to which individuals and groups will commit to personal responsibility for safety; act to preserve, enhance and communicate safety information; strive to actively learn, adapt and modify (both individual and organizational) behavior based on lessons learned from mistakes; and be held accountable or strive to be honored in association with these values.”

Establishing exceptional organizational safety culture is vital, as it directly affects performance and profit (Butler, 2016). These criteria emphasize that organizational safety culture is not only laws and regulations to be followed, but also an overall dynamic that concerns the well-being and productivity of individuals and groups. Safety culture therefore needs appropriate flows of information that allows all employees to be up-to-date and be part of a shared culture of safety. Safety culture does not only include the transmission of information, but also creation of information and values through exchanges amongst organization members. Communication is necessary for safety culture to properly exist. If communication is the mechanism through which safety is transmitted and created, then all the individuals who communicate with the organization are key to the organization's creation of its own safety culture, which ultimately influences employees' behaviors.

Technostress is a manifestation of a lack of safety culture. It is evident that any intervention to prevent or minimize technostress begins with the recognition that it is a factor which affects performance within the organization. Following recognition of technostress, it is possible to focus on work, technological, and communicative processes involved in this emerging risk.

## A Proposal: Positive Technology for Technostress Prevention and Management

While there have been several attempts in organizations to counteract techno-stressors (Dello Iacovo, 2012; Tarquini, 2014), previous attempts were neither anchored in any theoretical framework nor preventive. Rather, such attempts were compensative, and their effectiveness was highly anecdotal. A scientific approach proven to be highly effective in producing positive change is Positive Psychology (Seligman and Csikszentmihalyi, 2000; Seligman, 2002), with its derivative Positive Technology (Calvo and Peters, 2012; Riva et al., 2012, 2014). Positive Psychology postulates that personal experiences can be leveraged to foster well-being and personal growth. Similarly, Positive Technology is “the scientific and applied approach to the use of technology for improving the quality of our personal experience” (Riva et al., 2012, pp. 70). Perceived quality of personal experience occurs at three different domains: hedonic (technology is used to generate positive experiences); eudaimonic (technology is designed to support individuals in reaching “engaging and self-actualizing experiences”); and social/interpersonal (technology helps improve connectedness between individuals or groups).

**Figure 1:**
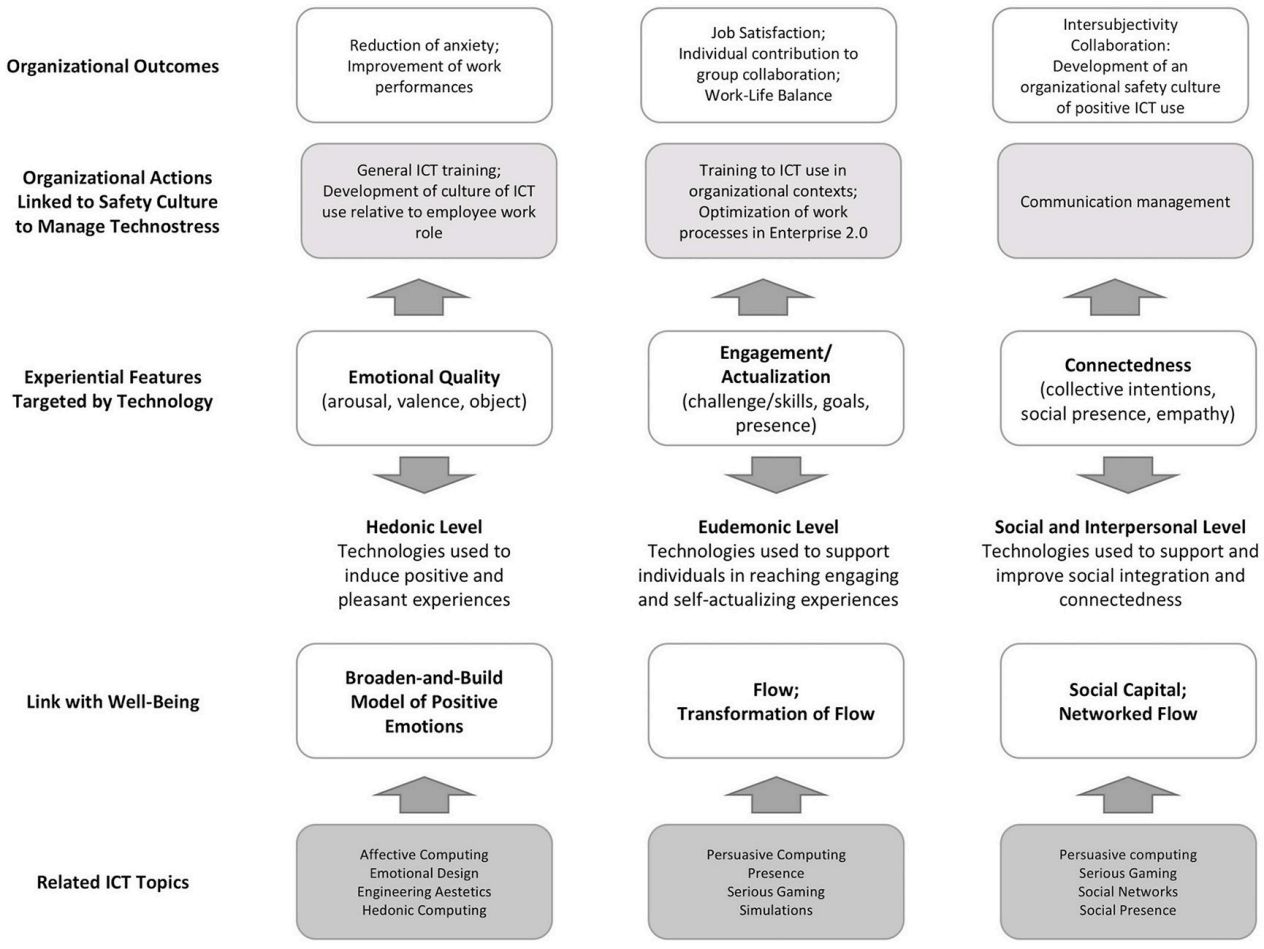
Positive Technology domains and their application to organizational safety culture (adapted from Riva et al., 2012).

At the bottom of Figure 1, Positive Technology domains are shown, as designed by Riva et al. (2012) (bottom part of the figure). At the top of Figure 1, the corresponding action of organizational culture safety are shown. These actions can generate positive experiences in companies and minimize technostress. It is possible to note that organizational cultural actions mediate between organizational outcome and the use of technologies. All three domains of personal experience affected by Positive Technology are as follows:

- Hedonic: at the individual level, positive emotions can be induced if the technologies are well designed and compatible to the employee's role within the company. An organization that employs this approach to technologies and work processes may develop a Positive Technology-based culture that prevents technostress. Employees using positive technologies could experience a reduction of techno-anxiety—as the ICTs are build according to their specifications, abilities, and needs—and perceive the work requests as fitting their role and way of working, therefore avoiding techno-overload, that is the amount of information received by the employees is appropriate to their roles and work processes. Reduced levels of anxiety and having more role-appropriate tasks may positively affect work performances.
- Eudaimonic: a technology within a work setting can generate an effect at this level if it is designed correctly, and employee training is performed accordingly. The key for a eudemonic experience is balancing employees' abilities with the technology that supports the task to be completed. If complex ICTs take into consideration the workers and their needs, their design and implementation will be less traumatic and require less employee training and adjustments. Even the most complex ICT can be perceived as easy-to-use with sufficient training. If the task and the technology are more complex than the employee's training and abilities, the technology will be perceived as an extraneous imposition, and then techno-complexity (perceived effort required to understand technology), techno-insecurity (perceived threat of not being up-to-date with the ICTs required for the job), and techno-uncertainty (perceived instability in work processes) can occur, ultimately resulting in technostress. The transition to the Enterprise 2.0 model entails slowly leaving work processes related to Web 1.0 tools, such as email, and adopting 2.0 tools (such as social media, blogs, wikis). Such change can be the opportunity to assess current ICTs and related work processes to develop and optimize new ways of working and relative technologies. On the contrary, collaboratively designed, Positive Psychology-based work processes and technologies make the employees' job easier, more satisfying, and less stressful. Consequently, employees may take more active part to collaborative work processes. Well-designed processes and ICTs will not require extra time and effort from the employees, thus preserving their work-life balance, avoiding techno-invasion, techno-strain, and techno-addiction.
- Social/interpersonal: many organizations are moving toward systems that exploit collaborative intelligence processes (Lee and Lan, 2007), requiring their employees to communicate with each other to generate a competitive advantage. A well-designed positive technology and work processes must support social presence, that is, the perception that others are present in the same digital environment and have a specific intention or task (Triberti et al., 2018), and intersubjectivity, that is the process to reach mutual comprehension (Galimberti, 2011). At this level, it is important that individuals share the same set of rules and regulations about how, when, and what is appropriate to communicate. This type of rules makes up part of the organizational safety culture, as they define boundaries for proper ways of communicating, setting appropriate time and space for using the ICT (reducing techno-invasion), and type of information and effort required (reducing techno-overload), overall limiting techno-strain (perceived stress due to the use of technology). When designing positive work ICTs and their work processes, it is therefore paramount that they have an embedded communication management system (e.g., system do not forward emails to employee after the end of business hours) that respects and contributes to the developing of this organizational safety culture, and helps prevent technostress.

All the suggestions made above may be applied both in a preventive and a corrective way. Literature shows that the Positive Technology approach rarely has been applied preventively, but the preventive perspective may prove to have more impactful and lasting effects, both on employees and organizations, as it can be easily included in an organization's safety culture. Bacchini (2014) states that organizational safety culture should become the general corporate organizational culture, as it respects the employees, it abides to laws and regulations, and it improves business performance, all the while promoting health and safety. As technology is essential to any company nowadays, adopting a Positive Technology perspective in designing not just the technologies themselves, but also the work processes, is the first step to prevent technostress and its related techno-stressors, and enduring happy, healthy, and satisfied employees, and an efficient and productive organization.

## Conclusion

This article was an opinion piece aimed to present technostress as a new field for the Positive Technology approach, which has only recently been applied to real-world contexts. Positive Technology can be considered as a proactive solution for organizations and companies who seek to increase their employees' well-being and prevent technostress. In particular, Positive Technology-designed solutions highlights how the three main dimensions of techno-stress and its other stressors may be reduced, or even eliminated, if this approach is used as a thinking framework for the organization.

This perspective is not without limitations, as some ICTs found within companies cannot be designed to stimulate the hedonic, eudemonic, and social/interpersonal levels of personal experience separately. All the levels contribute to employees' well-being and other organizational outcomes, as well as to prevent technostress. Positive Technology experts can contribute to the design of such technologies and related work processes, and interventions directed toward preventing and managing technostress. Another limitation is that this framework currently remains theoretical and requires implementation and observation in the field. Field research is possible, but may pose challenges, such as the time constrains demanded by design and implementation within an organization, which may not completely support the research requirements.

Conditions necessary for this approach to work is for companies, their employees, and Positive Technology experts to work together in designing new ICTs or modifying existing systems to include work processes that support such technologies. Without appropriate collaboration, technologies will induce technostress, rather than preventing it. As for any technology or process introduced within an organization, Positive Technology must be designed according to the organizational safety culture to which it will belong and contribute.

## Author Contributions

EB, FG, and CG conceived the presented ideas and participated to the literature review search. EB and CG wrote the manuscript; FG, IV, CRM, CR, and AS helped with the literature search and revised the first draft of the manuscript. EB acted as corresponding author.

### Conflict of Interest Statement

The authors declare that the research was conducted in the absence of any commercial or financial relationships that could be construed as a potential conflict of interest.
